# ELS (Ethical Life Support): a new teaching tool for medical ethics

**DOI:** 10.1186/s13054-019-2474-x

**Published:** 2019-06-06

**Authors:** Marco Vergano, Giuseppe Naretto, Fabrizio Elia, Enrico Gandolfo, Chiara Nebris Calliera, Giuseppe R. Gristina

**Affiliations:** 1 Department of Anesthesia and Intensive Care, San Giovanni Bosco Hospital, P.za del Donatore di Sangue, 3, 10154 Turin, Italy; 2 High Dependency Unit, San Giovanni Bosco Hospital, Turin, Italy; 3 Emergency Department, Martini Hospital, Turin, Italy; 4 Department of Public Health and Pediatrics, School of Nursing, University of Turin, Turin, Italy; 5 Bioethics Working Group, Italian Society of Anesthesia and Intensive Care Medicine (SIAARTI), Rome, Italy; 6 Ethics Committee, Italian Society of Anesthesia and Intensive Care Medicine (SIAARTI), Rome, Italy

The curriculum of critical care and emergency clinicians is usually packed with basic and advanced *life support* courses: cardiovascular emergencies, trauma, pre-hospital care, pediatric emergencies, and extra-corporeal life support are just a few examples of the most common topics.

Overall, these courses are not only well settled and familiar to physicians and nurses: they usually provide an excellent training opportunity, improving both knowledge specific to the field of interest and technical skills, through clinical scenarios, high fidelity simulations and hands-on sessions with medical devices. Moreover, the standardization of the training and of the evaluation process—both for students and for instructors—and a solid groundwork of constantly updated international best practice guidelines ensure not only the quality of the educational intervention but also the building of a common language that can be easily shared among colleagues across the world.

**Fig. 1:**
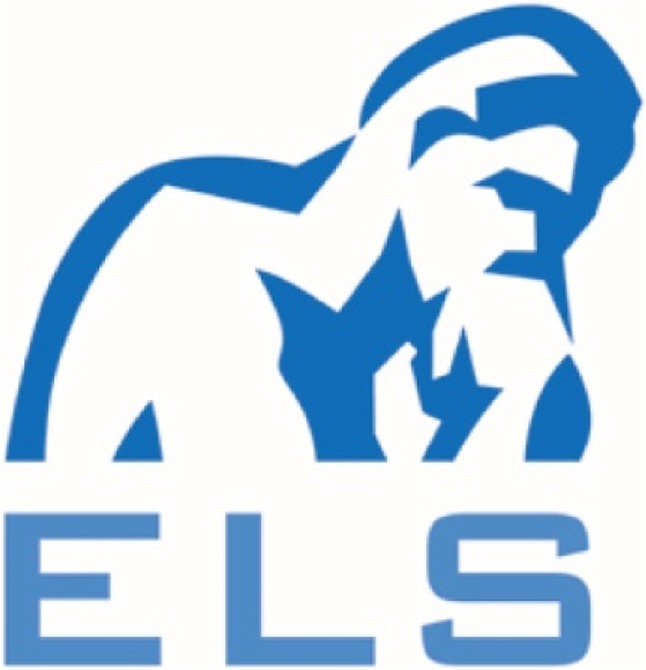
ELS logo

Sharing a common ground of daily exposure to clinical cases, passion for clinical ethics and end-of-life care, and a few years’ experience in studying and teaching ethical issues in different settings, a couple of years ago, we started developing an “ELS—Ethical Life Support” project: a short but comprehensive manual and a 1-day highly interactive course (Fig. 1).

Obviously, our aim has never been to compress a full ethics program in a short reading and a single-day course, rather to provide basic ethical reasoning abilities and vocabulary, some practical communication skills and basic conflict management strategies.

The whole idea behind our project has never been to train bioethicists *by profession*, rather to address bioethical issues *in the profession*.

A first Italian edition of the manual [1] was published in 2018 and, shortly after, the first official “ELS—Ethical Life Support Basic Course” took place. In the following months, the project earned the joint endorsement of the Italian Society of Anesthesia and Intensive Care Medicine (SIAARTI) and of the Italian Society of Emergency Medicine (SIMEU).

In order to maintain the similarity of our project to other *LS courses, we developed an ABCD structure, translating the usual Airway–Breathing–Circulation–Disability sequence into an Acknowledge–Be aware–Communicate–Deal approach, as shown in Table 1.

**Table 1:** ABCD sequence

Section	Learning objectives	Topics/tools
A—*Acknowledge*	• Identifying the ethical issue • Facts vs values • Ethics and healthcare ethics	• Introduction and agenda • The *trolley problem* and variations (lecture, discussion)
B—*Be aware*	• Defining boundaries • Assessing roles and responsibilities: who decides?	• Overview of the law and professional self-regulation (lecture and discussion) • Two short clinical cases (group discussions and role-playing)
C—*Communicate*	• Acknowledging the *ownership* of *space*, *time*, and *language* • Fostering good communication and respect	• Short videos, lecture, and discussion • Small group exercises • Role-playing
D—*Deal*	• Managing complexity • End-of-life decisions • Tolerating uncertainty	• Medical errors, conflicts, and disagreement (lecture and discussion) • Multi-step clinical case (small group discussion) • Take-home messages

The purpose is to guide the student from theory to practice, from the identification of the ethical component—often unexamined—to the management of dilemmas and conflicts, through the acquisition of practical skills.

While the majority of the information is provided by the manual and is verified through a quiz test at the beginning of the course, the training day is very interactive and includes clinical cases, videos, role-playing, small group discussions, and exercises.

Section A (*Acknowledge*) provides the groundwork of ethical reasoning in a medical setting, showing how to separate facts from values, normative from descriptive judgments, and *gut feelings* from rational ideas. It helps focus on the patient’s *biography* rather than merely on his/her *biology* [2], and thus avoid automatic decisions, in order to protect the patient from a *conveyor-belt medicine* approach [3]. A short overview of different ethical theories and different approaches in medical ethics is also provided.

Section B (*Be aware*) is intended to show to which extent our field of action is defined by professional deontology and by the law, that shape the boundaries of our practices as well as the roles and responsibilities of all the actors involved in a clinical case. The “who decides?” issue ranges from the disagreement with a competent patient to the role of family members as contributors to substitute decision-making [4] for an unconscious patient, a common scenario in the ICU.

Section C (*Communicate*) emphasizes the need to train in communication skills in the same way it happens with other professional skills. It underlines the usual asymmetry of medical communication, when fragile and vulnerable patients face clinicians, who are the usual *owners* of all the three components of communication—*space*, *time*, and *language*. It also recognizes the central role of communication in the ICU, as a key tool for humanization [5] and respect [6].

Section D (*Deal*) explores some of the most common challenges clinicians face in everyday practice, from prognostic uncertainty to end-of-life decisions, from medical errors to value disagreement. The purpose is to provide basic tools to manage complexity and tolerate uncertainty [7], reducing conflicts and *moral distress*. An example of one of the teaching tools is shown in Table 2.

**Table 2:** An example of one of the teaching tools

The final part of the course is a challenging three-step clinical case, with different sequential ethical dilemmas. A young girl is in need of an urgent liver transplant following a voluntary alcohol and paracetamol intoxication. Her advanced directives and her parents’ will must be taken into account, while caring for her from admittance to the emergency department, through the general ward, to a long stay in the ICU. Each step is analyzed through small group discussions, following the ABCD approach; at the end of each step, participants are asked not only to argue their own choices, but also to defend the opposite positions. Regardless of individual participants’ values, the focus is on the importance of end-of-life shared decision-making, taking into account the best available scientific evidence as well as the best reconstruction of the patient’s preferences [8].	

After a half dozen editions (approximately 30 participants each), with slight but continuous improvements, the feedback we are receiving confirms that the formula is pretty effective in achieving our initial goal.

We are also realizing that—through the interactive approach and given the unique background and personality of each participant—the multi-disciplinary and multi-professional composition of the class adds a valuable contribution to each edition, building what we might consider a real process of *peer education*.

The differences between our project and the rest of *LS courses are evident. As Julian Savulescu clearly described, “Science is about the way the world is, was, will be, could be, would be. Ethics is concerned with norms and values. […] It is about good and bad, right and wrong. Ethics is about values; science is about facts” [9]. No guideline will ever be able to define the best course of action, when dealing with ethical issues and value-laden judgments, so a shared *ethos* may remain an unattainable goal in a pluralistic society.

Nevertheless, we strongly believe that the acquisition of a common stepwise approach and a shared vocabulary—through a simple and easily reproducible educational tool—may foster a culture of mutual respect among colleagues and search for appropriateness.

This is why we would like our project to remain open and continue to grow through critiques, proposals, and contributions.

Good clinical practice requires better medical ethics and innovative teaching tools.
